# Free Functional Latissimus Dorsi Reconstruction of the Quadriceps and Hamstrings following Oncologic Resection of Soft Tissue Sarcomas of the Thigh

**DOI:** 10.1155/2021/8480737

**Published:** 2021-12-08

**Authors:** Matthew T. Houdek, Elizabeth P. Wellings, Katherine E. Mallett, Rachel L. Honig, Peter S. Rose, Steven L. Moran

**Affiliations:** ^1^Mayo Clinic, Department of Orthopedic Surgery, Rochester, MN, USA; ^2^Mayo Clinic Division of Plastic and Reconstructive Surgery, Rochester, MN, USA

## Abstract

**Background:**

Limb-salvage surgery combined with radiotherapy has become the primary treatment for soft tissue sarcomas of the extremity. Free functional latissimus flaps (FFLF) are an option to restore function in the setting of volumetric muscle loss. The purpose of the current study was to examine the use of FFLF in patients undergoing resection of thigh sarcoma.

**Methods:**

Twelve patients with a sarcoma involving the hamstring (*n* = 6), quadriceps (*n* = 5), or combined (*n* = 1) defects which included multiple muscle groups were reviewed. This included 9 males and 3 females with a mean age and body mass index of 56 ± 12 years and 31.3 ± 5.7 kg/m^2^.

**Results:**

The mean defect volume and operative time was 3,689 ± 2,314 cm^3^ and 587 ± 73 minutes. Following reconstruction, the mean knee range of motion (ROM), MSTS93 score, and muscle strength was 89 ± 24°, 90 ± 15%, and 4 ± 1; with 75% of patients ambulating without gait aids. Seven (58%) patients sustained a complication, namely, delayed wound healing (*n* = 2).

**Conclusion:**

Although there was a high incidence of complications, FFLF can restore active knee ROM and function, with most patients ambulating without gait aids following reconstruction of large oncologic defects in the thigh.

## 1. Introduction

Advances in surgical techniques and the addition of neo adjuvant or adjuvant radiotherapy have allowed limb-salvage surgery to become the primary means of treatment for patients with nonmetastatic soft tissue sarcomas of the extremities [1]. Although the margin of resection between the tumor and preserved critical structures, such as nerves and blood vessels, can be ≤1 mm, the goal for a margin of resection in muscle or subcutaneous tissue is often 2 cm [2]. Following resection of a primary soft tissue sarcoma, or in the setting of a reexcision of a previous inadvertent excision, reconstructive surgeons are often faced with volumetric muscle loss, with limited options for functional reconstruction [3–5].

Reconstruction of the soft tissue defect should provide obliteration of the potential space in addition to restore the functional loss associated with the resection [6–8]. Although free muscle flaps have been found to adequately provide wound coverage and are safe in the setting of radiotherapy [4], few studies have examined the function and motion of the extremity following reconstruction. Following traumatic injuries to the upper and lower extremity, free functional muscle flaps have become popular to restore function [9–12]; however, there are limited data on the use of these flaps in the oncologic setting to provide coverage with potential space obliteration, in addition to a functional reconstruction [13]. The purpose of this study was to review our institutions experience with free functional latissimus dorsi muscle flaps to restore either quadriceps or hamstring function following oncologic resection.

## 2. Patients and Methods

Following institutional review board approval, we performed a retrospective review of patients undergoing free functional latissimus reconstruction of the thigh following resection of a soft tissue sarcoma at our institution between 2018 and 2019. The study group (Table 1) consisted of 12 patients with soft tissue sarcoma which either involved the hamstrings (*n* = 6, 50%), quadriceps (*n* = 5, 42%), and combined quadriceps and hamstrings (*n* = 1, 8%). There were 9 males (75%) and 3 females (25%), with a mean age and body mass index (BMI) of 56 ± 12 years and 31.3 ± 5.7 kg/m^2^ at time of surgery. Primary tumor resection was carried out in 7 patients (58%), while 5 patients (42%) underwent reexcision following an inadvertently excised tumor. Tumor grade was classified as high (*n* = 9, 75%) and low (*n* = 3, 25%) grades, with all tumors being deep to the fascia. Tumor histology included undifferentiated pleomorphic sarcoma (UPS, *n* = 4, 33%), myxoid liposarcoma (*n* = 3, 25%), dedifferentiated liposarcoma (*n* = 2, 17%), myxofibrosarcoma (*n* = 2, 17%), and extraskeletal osteosarcoma (*n* = 1, 8%). As part of their multidisciplinary care, all patients received preoperative radiotherapy, at a dose of 50 Gy, with surgical resection occurring 3–5 weeks following the completion of radiotherapy.

### 2.1. Patient Follow-Up

Patients were followed longitudinally every 3 months postoperative for the first 2 years and then every 6 months for years 2–5 postoperative for local recurrence or distant disease. Surveillance included a CT scan of the lungs and a contrast-enhanced MRI of the extremity. All patients had achieved at least 1-year of clinical follow-up, with the mean follow-up of 2 years (range 1–3 years). At each visit, the Musculoskeletal Tumor Society (MSTS93) [14] is calculated. Range of motion of the knee was measured in degrees. Active motion was recorded against gravity, in patient with quadriceps reconstruction performed in the seated position and for hamstring reconstruction in the prone position. Muscle strength was graded 0–5: 0, no evidence of flap contraction; 1, muscle contraction but no joint motion; 2, poor strength and motion when gravity is eliminated; 3, fair strength against gravity alone; 4, good strength against gravity and some resistance; and 5, normal strength capable of full motion of the knee joint and against maximal resistance.

### 2.2. Statistical Analysis

Disease-specific survival and recurrence free survival were determined using the Kaplan–Meier model. Continuous variables were compared using Student' *t*-tests, and categorical variables were compared using Fisher exact tests where appropriate. A *p* value of <0.05 was considered significant.

## 3. Results

### 3.1. Operative Technique

**Figure 1:**
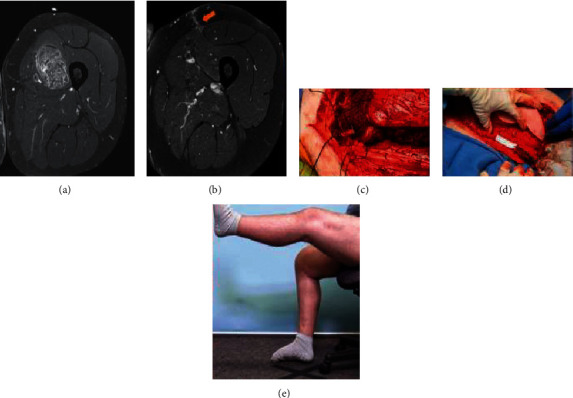
Axial MRI with contrast (a) showing a deep soft tissue mass which was inadvertently excised. The pathology was consistent with sarcoma, and on postexcision MRI (b), there was evidence of the surgical incision (arrow) which involved the sartorius, rectus femoris, and vastus medialis and intermedius. During the resection of these muscle groups, care is taken to identify the nerve fibers entering the muscle bellies which are tagged (ties) for repair (c). The free functional latissimus with a skin paddle was inset with care to be sure the resting tension was correct (d). Following surgery, the patient had active knee extension (e) and ambulated without gait aids.

**Figure 2:**
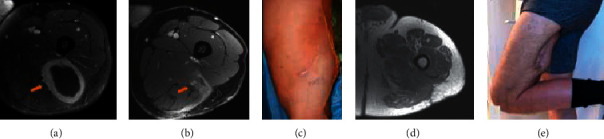
Axial MRI with contrast (a) showing a soft tissue sarcoma, involving the posterior compartment of the thigh abutting the sciatic nerve (arrow). The patient underwent a nononcologic resection at an outside center, and following the excision axial MRI with contrast (b) shows residual tumor abutting the sciatic nerve (arrow) and soft tissue edema involving the posterior compartment. In addition, they had an incision and surgical drain placed which did not follow oncologic principles (c). The resection included the sciatic nerve and the lateral hamstring complex and semimembranosus (d) with reconstruction utilizing a free functional latissimus with a skin paddle. Following surgery, the patient had full knee extension with flexion past 90° (e) ambulating without gait aid.

All resections and reconstructions were performed by the same orthopedic oncology and plastic and reconstructive team. Patients are placed in either a rolling lateral position which allows access to the entire lower extremity and ipsilateral latissimus flap harvested (hamstring resections) or in the supine position with the contralateral latissimus flap harvested (quadriceps reconstructions). The oncologic resection is performed prior to harvest of the latissimus flap with the goal to achieve a negative margin. The size of the resection was dependent on the tumor location on immediate preoperative MRI. For anterior thigh resection (Figure 1), the resection included the entire quadriceps complex (*n* = 2); rectus femoris, sartorius, vastus medialis, and vastus intermedius (*n* = 2); and rectus femoris, vastus medialis, and vastus intermedius (*n* = 1). For posterior thigh resections (Figure 2), the resection included the entire hamstring compartment (*n* = 2); entire hamstring and adductor magnus (*n* = 1); semimembranosus and biceps femoris (*n* = 1); semimembranosus, semitendinosus, and adductor magnus (*n* = 1); and biceps femoris (*n* = 1). One patient had a combined anterior and posterior tumor, and the resection included the biceps femoris, vastus lateralis, vastus intermedius, and rectus femoris. For this patient, the functional reconstruction was for the quadriceps defect. One of the hamstring resections included removal of the sciatic nerve due to a previous inadvertent tumor excision done elsewhere. Negative margins were obtained in all cases. The mean tumor size and volume on the resected specimen were 12 ± 7 cm and 1,074 ± 1,145 cm^3^, respectively. The mean total defect size (maximum dimension of composite tissue resected) was 26 ± 7 cm, with a mean total volume of resection of 3,689 ± 2,314 cm^3^. The mean operative time was 587 ± 73 minutes, of which the mean time for the reconstruction was 369 ± 89 minutes.

During the oncologic extirpation, care is taken to identify the perforating nerve branches entering either the quadriceps or hamstring muscle bellies at the proximal level of resection as the nerves come from either the sciatic or femoral nerve. These are tagged and cut as long as possible sharply for subsequent repair. The latissimus flap is harvested in the standard fashion, and care is taken to preserve the thoracodorsal nerve. The entire latissimus dorsi including the tendonous attachment is exposed, and before releasing the muscle, the natural resting tension is marked at 5 cm intervals. The recipient site is prepared prior to ligation of the pedicle to the latissimus. Suitable vascular pedicles and nerve branches are identified, and then, the latissimus pedicle is ligated, and the latissimus is inset. The tendonous portion of the latissimus is attached to the distal portions of the cut muscle bellies at the level of their tendonous insertion. The muscular portion of the latissimus can be either directly sutured to the cut ends of the muscle bellies or attached to the femur or ischium with suture anchors. Muscle to muscle repair was aided with the use of the modified Krakauer suture technique and the use of acellular dermal matrix or Achilles allograft. The muscle is temporally inset to allow for the microvascular anastomosis and for the nerve repair which is done as closely to the muscle belly as possible. Once the microvascular procedures are completed, the resting tension is then adjusted to allow for the markings on the latissimus to measure 5 cm [10]. All flaps were harvested with a skin island for flap monitoring; however, in all patients, an implantable Doppler (Cook-Schwartz, Cook Medical, Bloomington, IN) was used for flap monitoring. The wounds were then closed in multiple layers, and in 5 (42%) patients, an additional skin graft was needed for wound closure. None of the reconstructions were supplemented with additional lower extremity muscle transfers or tendon transfers.

Patients were placed in a postoperative bulky splint with the leg in extension for quadriceps reconstructions and in slight flexion for hamstring reconstruction. Patients were kept on bed rest for 5 days, and then, a progressive dangling protocol is started. All patients are kept on pharmacological DVT prophylaxis for 28 days. They are kept toe-touch weight bearing for 6 weeks, with progression of their weight bearing dependent on wound healing. Range of motion of the knee is held until 3 months postoperative, at which time passive, active-assisted, and active range of motion are started.

### 3.2. Group Comparison

There was no difference in the mean patient age, proportion of males, high-grade tumor histology, mean resection size, and volume (*p* > 0.05) between patients who underwent reconstruction of the quadriceps or hamstrings (Table 2).

### 3.3. Oncologic Outcome

There were no cases of local tumor recurrence; however, 3 patients developed metastatic disease (2 patients with lung metastatic disease and 1 patient with soft tissue metastatic disease). Metastatic disease leads to death due to disease in 1 patient, and in the remaining, 2 patients had undergone resection of their metastatic disease and one is currently disease free and the other has had progressive disease. The 2-year metastatic free and disease-specific survival were 70% and 80%. Two cases of metastatic disease occurred in patients with high-grade tumors, and one case occurred in a patient with a low-grade tumor.

### 3.4. Surgical Complications and Functional Outcomes

Recipient site complications occurred in 7 (58%) patients and included hematoma (*n* = 2), delayed wound healing (*n* = 2), lymphedema (*n* = 1), and deep venous thrombosis (*n* = 1). These complications resulted in reoperation in 2 patients to debride postoperative hematoma. There were no cases of flap loss. Donor-site complications occurred in 1 patient, including hematoma which required irrigation and debridement. Due to the extent of periosteal stripping in the setting of preoperative radiotherapy, 5 patients underwent prophylactic stabilization of the femur using an intramedullary nail.

Following reconstruction, the mean MSTS93 score was 90 ± 15% (Table 3), with no difference in the mean MSTS93 score between patients with a reconstruction for the quadriceps or hamstring (85 ± 19% vs. 96 ± 5%, *p*=0.22). The mean total knee range of motion (ROM) postoperatively was 89 ± 24°, with no difference in the mean knee ROM between patients with a reconstruction for the quadriceps or hamstring (93 ± 30° vs. 84 ± 20°, *p*=0.54). All patients were ambulatory at most recent follow-up. Three patients needed a cane to assist with ambulation; in addition, one patient required ankle-foot-orthosis (AFO). All patients regained the ability to move the reconstructed muscle group against gravity. The mean muscle strength as graded from 0 to 5 using the manual muscle testing grading system was 4 ± 1, with no difference in the mean postoperative strength between patients with a reconstruction for the quadriceps or hamstring (3.5 ± 1 vs. 4 ± 0, *p*=0.17).

## 4. Discussion

The thigh is the most common location for soft tissue sarcomas requiring resection of the muscles of the anterior or posterior compartment. Such oncologic resections can impart substantial functional limitations for patients. In the setting of massive volumetric muscle loss, options to restore the function are limited, and the results of the current study highlight the potential utility of a free functional latissimus flap to not only provide soft tissue coverage and obliterate the potential space from the surgical resection but also the ability to restore function of the resected compartment.

Quadricep reconstruction utilizing a free functional latissimus flap is best indicated if a portion of the muscle compartment can be spared [6]. It has been shown the isometric strength of the quadriceps decreases with each component resected and has an impact on the functional outcome of patients [15]. In cases where the entire compartment is excised, the latissimus has difficulty compensating for the loss in function secondary to the latissimus being 1/3 the size of the quadriceps complex [6]. In order to compensate for the lack of bulk in these situations, the use of additional pedicle muscle flaps can assist the latissimus in functional reconstruction [6, 16]. In the current series, the two cases which had a fair outcome for quadriceps reconstruction included a patient where the entire quadricep complex was resected and in a patient where the lateral hamstring and quadriceps were resected. Although a pedicled muscle flap could have potentially assisted in reconstruction in one of the patients (total quadricep resection), in the other patient, the use of the lateral hamstring to reconstruct the lateral quadriceps as proposed by Pritsch et al. [16] would have been impossible secondary to it being resected with the tumor specimen. However, it should be noted that the use of pedicle muscle flaps from the hamstring to help restore knee extension and does result in a loss 28–67% of knee flexion strength [15]. As such, reconstructive surgeons need to take into account the potential loss of hamstring function in order to restore quadriceps function with these pedicle flaps and balance the benefit of the additional muscle for knee extension strength to the loss of flexion.

Unlike the quadricep reconstruction, there are limited data on the use of a free functional latissimus flap to restore knee flexion strength following resection of a soft tissue sarcoma [13]. In a series by Doi et al. [13], the authors reported on 4 patients who underwent functional reconstruction using a free latissimus flap. In this series, all had restoration of at least antigravity knee flexion strength, which is similar to the results of the 6 patients in the current series. Removal of the entire hamstring compartment results in considerable deficits in knee flexion strength and moderate patient impairment [15]; however, the results of the current series highlight the restoration of knee flexion strength with a free functional latissimus flap. It is likely that patients had improved MSTS93 scores in the hamstring reconstruction group, although this was not significant, secondary to the preservation of the gastrocnemius muscles which can also assist with knee flexion; however, the main assistance for knee flexion is during the first 15° [17]. The results of the current series show the addition of a free functional latissimus flap can improve knee flexion and functional outcome, with a majority of patients able to ambulate without the assistance of gait aids.

The rate of complications following free functional latissimus flaps has reported to be high. In the series by Innocenti et al. [6], there was a 63% rate of complications, namely, due to femur fracture which was associated with periosteal stripping, use of bone anchors in the femur, and radiotherapy. Radiation-associated fractures have been found to be more common in the femur and associated with higher doses of radiotherapy [18, 19]. In order to reduce the risk of radiation-associated fractures, prophylactic stabilization of the femur has been recommended [20]. In order to potentially reduce the risk of fracture, in the current series, we utilized prophylactic stabilization at 3-month postoperative in the setting of preoperative radiotherapy, periosteal stripping, and use of soft tissue anchors in the femur and so far, have not had a patient sustain a radiation-associated fracture.

Although it is one of the most common flaps harvested, the harvest of the latissimus is not without the potential reduction in the loss of shoulder function long term [21, 22]. The latissimus works in conjunction with the teres major and the pectoralis major to stabilize the shoulder and to adduct, rotate, and extend the joint [22]. If the teres major and the pectoralis major are maintained, the loss of the latissimus is made up for by these muscles. Therefore, although there are some biomechanical changes present, they do not substantially impact function nor shoulder range of motion. Instead patients should be cautioned on the risk of fatiguing sooner in the shoulder with the use of overhead activities such as climbing ladders, painting, and swimming [22].

The results should be interpreted considering certain limitations. Although this is a relatively large series of free functional latissimus flaps used for thigh reconstruction, the small sample size limits our statistical analysis and the comparison we can perform. Each resection is different and determined by the tumor location and previous operations, which dictated the tissue resection imparting heterogeneity in the extent of resection; however, all resections were performed by the same orthopedic and plastic and reconstructive teams. During the treatment time, other reconstructive options such as pedicle flaps are available, and there was no comparison group included in the current series. To analyze muscle strength, we utilized manual testing, and formal testing with EMG was not performed.

This study shows the utility of free functional latissimus flaps to provide coverage and restoration of knee range of motion following resection of a soft tissue sarcoma of the thigh. Although these procedures are technically demanding, they allow for most patients to ambulate without gait aids and to return to all activities of daily living who would otherwise have substantial impairment in function of the extremity.

## Figures and Tables

**Table 1:** Patients undergoing free functional latissimus flap reconstruction following resection of thigh soft tissue sarcoma.

Patient	Age (years)	Gender	Histology	Previous surgery	Muscles resected	Volume resected (cm^3^)	Postoperative knee ROM (°)	Postoperative MSTS93 score (%)	Muscle strength
1	55	Male	Extraskeletal osteosarcoma	Yes	Semimembranosus	7079	100	97	4
					Long head biceps femoris				
					Short head biceps femoris				
					Sciatic nerve				
2	37	Male	UPS	No	Entire hamstring complex	856	90	100	4
3	39	Male	Myxoid liposarcoma	No	Entire hamstring complex	4805	45	93	4
4	62	Male	Dedifferentiated liposarcoma	No	Semimembranosus	7846	90	100	4
					Semitendinosus				
					Adductor magnus				
					Adductor longus				
					Gracilis				
5	53	Male	Myxoid liposarcoma	No	Entire hamstring complex	4118	90	97	4
6	71	Male	Myxofibrosarcoma	Yes	Long head biceps femoris	3828	90	87	4
					Short head biceps femoris				
7	66	Female	Myxoid liposarcoma	No	Vastus lateralis	3413	45	53	3
					Long head biceps femoris				
					Short head biceps femoris				
8	62	Male	UPS	No	Rectus femoris	5297	115	100	4
					Vastus medialis				
					Vastus intermedius				
9	50	Female	UPS	Yes	Entire quadriceps complex	1120	70	70	2
10	47	Male	Dedifferentiated liposarcoma	Yes	Rectus femoris	3223	110	100	4
					Vastus medialis				
					Vastus intermedius				
					Sartorius				
11	75	Male	Myxofibrosarcoma	Yes	Rectus femoris	1627	100	90	4
					Vastus medialis				
					Vastus intermedius				
					Sartorius				
12	52	Female	UPS	No	Entire quadriceps complex	1053	120	97	4

^*∗*^ UPS, undifferentiated pleomorphic sarcoma; ROM, range of motion; MSTS93, musculoskeletal tumor society score.

**Table 2:** Comparison of patients undergoing free functional muscle reconstruction of the thigh.

Demographic	All patients (*n* = 12)	Quadriceps reconstruction (*n* = 6)	Hamstring reconstruction (*n* = 6)	*P* value
Mean patient age	56 ± 12 years	59 ± 11 years	53 ± 13 years	0.42
Male gender	9 (75%)	3 (50%)	6 (100%)	0.18
High grade tumor	10 (83%)	5 (83%)	4 (67%)	1.0
Maximum resection size	26 ± 7 cm	23 ± 5 cm	30 ± 6 cm	0.07
Resection tumor volume	3,689 ± 2,314 cm^3^	2,622 ± 1,664 cm^3^	4,755 ± 2,506 cm^3^	0.11

**Table 3:** Functional outcome of patients undergoing free functional muscle reconstruction of the thigh.

Outcome	All patients (*n* = 10)	Quadriceps reconstruction (*n* = 6)	Hamstring reconstruction (*n* = 6)	*P* value
Mean postoperative knee ROM	89 ± 24°	93 ± 30°	84 ± 20°	0.54
Mean postoperative MSTS93 score	90 ± 15%	85 ± 19%	96 ± 5%	0.22
Use of gait aid	3 (30%)	2 (33%)	1 (17%)	1.0
Mean muscle strength	4 ± 1	3.5 ± 1	4	0.17

## Data Availability

The data used to support the findings of this study cannot be shared.
